# Superoxide dismutase A antigens derived from molecular analysis of sarcoidosis granulomas elicit systemic Th-1 immune responses

**DOI:** 10.1186/1465-9921-9-36

**Published:** 2008-04-25

**Authors:** Shannon S Allen, Whitney Evans, James Carlisle, Rana Hajizadeh, Michele Nadaf, Bryan E Shepherd, David T Pride, Joyce E Johnson, Wonder P Drake

**Affiliations:** 1 Department of Medicine, Division of Infectious Diseases, Vanderbilt University School of Medicine, Nashville, TN, USA; 2 Department of Biostatistics, Vanderbilt University School of Medicine, Nashville, TN, USA; 3 Department of Medicine, Division of Infectious Diseases, Stanford School of Medicine, Palo Alto, CA, USA; 4 Department of Pathology, Vanderbilt University School of Medicine, Nashville, TN, USA; 5 Department of Microbiology and Immunology, Vanderbilt University School of Medicine, Nashville, TN, USA

## Abstract

**Background:**

Sarcoidosis is an idiopathic granulomatous disease with pathologic and immunologic features similar to tuberculosis. Routine histologic staining and culture fail to identify infectious agents. An alternative means for investigating a role of infectious agents in human pathogenesis involves molecular analysis of pathologic tissues for microbial nucleic acids, as well as recognition of microbial antigens by the host immune system. Molecular analysis for superoxide dismutase A (sodA) allows speciation of mycobacteria. SodA is an abundantly secreted virulence factor that generates cellular immune responses in infected hosts. The purpose of this study is to investigate if target antigens of the sarcoidosis immune response can be identified by molecular analysis of sarcoidosis granulomas.

**Methods:**

We detected sodA amplicons in 12 of 17 sarcoidosis specimens, compared to 2 of 16 controls (p = 0.001, two-tailed Fisher's exact test), and 3 of 3 tuberculosis specimens (p = 0.54). Analysis of the amplicons revealed sequences identical to *M. tuberculosis *(MTB) complex, as well as sequences which were genetically divergent. Using peripheral blood mononuclear cells (PBMC) from 12 of the 17 sarcoidosis subjects, we performed enzyme-linked immunospot assay (ELISPOT) to assess for immune recognition of MTB sodA peptides, along with PBMC from 26 PPD- healthy volunteers, and 11 latent tuberculosis subjects.

**Results:**

Six of 12 sarcoidosis subjects recognized the sodA peptides, compared to one of 26 PPD- controls (p = 0.002), and 6/11 PPD+ subjects (p = .68). Overall, 10 of the 12 sarcoidosis subjects from whom we obtained PBMC and archival tissue possessed molecular or immunologic evidence for sodA.

**Conclusion:**

Dual molecular and immunologic analysis increases the ability to find infectious antigens. The detection of Th-1 immune responses to sodA peptides derived from molecular analysis of sarcoidosis granulomas reveals that these are among the target antigens contributing to sarcoidosis granulomatous inflammation.

## Background

Sarcoidosis is a disease of unknown etiology, characterized pathologically by noncaseating granulomas that most commonly involve the lung, skin, lymph nodes and eyes [1]. Syndromes with similar pathologic and immunologic features to sarcoidosis such as chronic beryllium disease [2], hypersensitivity pneumonitis [3], and tuberculosis [4] indicate that granulomatous diseases may or may not have an infectious etiology. Recently, several reports from independent laboratories have associated mycobacteria with sarcoidosis immunopathogenesis. One report of molecular analysis of sarcoidosis granulomas revealed the presence of multiple mycobacterial genes [5]. Humoral immune responses to *Mycobacterium *katG and heat shock proteins by sarcoidosis subjects [6,7] as well as the detection of these proteins in sarcoidosis granulomas have been reported [6,8]. Most recently, a report of Th-1 immune responses to mycobacterial antigens from sarcoidosis subjects added further support to the hypothesis that mycobacterial antigens may have a role in sarcoidosis pathogenesis [9].

Superoxide dismutase A (sodA) is a virulence factor secreted in large quantities by pathogenic *Mycobacterium *and *Nocardia *species, converting oxygen free radicals generated by host macrophages to hydrogen peroxide [10]. *M. tuberculosis *encodes two superoxide dismutases (SOD), an iron-cofactored enzyme (sodA) and a copper-zinc enzyme (sodC) [11]. Strong immune responses are elicited against sodA in tuberculosis-infected mice [12]. Molecular analysis of the sodA gene identifies *Mycobacterium *species [13]. The purpose of this study is to assess if molecular analysis of sarcoidosis granulomas will reveal microbial virulence factors which can induce Th-1 immune responses in the same subjects. Taking advantage of the capability of molecular analysis of sodA to identify *Mycobacterium *species, as well as the ability of sodA to generate strong immune responses, we conducted molecular analysis for the presence of *Mycobacterium *sodA in sarcoidosis granulomas, followed by immunologic assessment for Th-1 immune responses to sodA peptides in the same sarcoidosis subjects.

## Methods

### Recruitment of study participants

This study was approved by the Vanderbilt University Institutional Review Board for human studies, and informed written consent was obtained from the study participant or their surrogates, if required. All sarcoidosis subjects from the available patient database of the Vanderbilt University Pulmonary Clinic or members of the Middle Tennessee Sarcoidosis Support Group were invited to participate in the study. For inclusion in this study, the following criteria were used for patients with sarcoidosis: 1) clinical features of acute respiratory illness accompanied by erythema nodosum, hilar adenopathy and arthritis [Lofgren's syndrome], or indolent progressive pulmonary decompensation associated with radiographic findings, such as hilar adenopathy, reticulonodular infiltrates, or pulmonary fibrosis); 2) histopathologic features had confluent noncaseating granulomas, well circumscribed within the surrounding tissue with a variable amount of peripheral lymphocytic infiltration [4]); 3) known microbial causes for granulomata had to be excluded (i.e., specimens were negative for microorganisms by hematoxylin and eosin (H&E), fungal, acid fast bacilli (AFB), and auramine-O stains and on routine bacterial, fungal, and AFB cultures). Frozen or paraffin-embedded archival specimens from 21 sarcoidosis subjects were obtained; four were excluded due to the lack of granulomatous involvement on the slide or pathology inconsistent with the diagnosis of sarcoidosis, which left 17 specimens for molecular analysis. Nineteen frozen or paraffin-embedded control specimens were obtained from the archives of Vanderbilt Pathology Department, or the Cooperative Human Tissue Network: three tuberculosis specimens and 16 negative control specimens of infectious and non-infectious etiologies. All sarcoidosis and control specimens had their diagnostic slides independently confirmed by a pathologist.

For the enzyme-linked immunospot assay (ELISPOT), we were able to collect blood on 12 of the 17 sarcoidosis subjects. Due to the Health Insurance Portability and Accountability Act of 1996 (HIPAA) regulations, we could not contact the negative or positive control subjects of whom we had archival tissues. We invited 26 PPD negative (PPD-) healthy volunteers to participate as negative controls for the ELISPOT analysis. The PPD- volunteers had undergone PPD testing by the Vanderbilt employee health services. Subjects with latent tuberculosis (PPD+) were invited as positive controls, and had written documentation of their PPD status with no evidence of active disease at the time of study enrollment. Approximately 60% of the sarcoidosis, PPD- and PPD+ subjects had participated in a previous investigation of immune responses to mycobacterial antigens [14].

### DNA extraction of archival tissue

For each sarcoidosis and control specimen, the original paraffin-embedded tissue block was retrieved from the archives if available. From each block, we cut a single 5 μm section that was stained with H&E to confirm the pathology and demarcate areas of granulomatous involvement; another ten 10 μm sections were cut, three of which were used for extraction of DNA, and the remaining seven sections were stored for future analysis. The specimens were randomly processed for slide preparation. For each section from patients with sarcoidosis and for the control specimens, granulomata were macrodissected and extracted with disposable surgical blades. For the control specimens without granulomata, all tissue from three 10 μm sections was used for DNA extraction.

For the frozen specimens, 25 mg of frozen sarcoidosis or control specimens were initially minced with sterile disposable surgical blades prior to overnight proteinase K digestion. For all specimens, DNA was extracted with the QIAGEN DNAeasy extraction kit (QIAGEN, Valencia, CA) according to the manufacturer's instructions, with the exception of the following changes: 1) 60 μL of proteinase K was used at a concentration of 20 mg/mL; 2) after the overnight digestion, 0.5 mm glass beads (Biospec Products, Inc., Bartlesville, OK) were added to the completely digested tissue, which was subsequently vortexed for 45 seconds; 3) the DNA was eluted with 100 μL of Buffer AE, instead of 200 μL.

Tissue dissection and DNA preparation were performed in a dedicated clean room, which was separate from the rooms used for PCR analysis and sequencing. The extracted DNA was stored at -80°C. Groups of tissue specimens from patients with sarcoidosis, tuberculosis, and controls were processed in parallel during all steps of the procedure, including extraction of the DNA, amplification and detection of mycobacterial DNA, and sequence analysis. The clinical diagnosis of each specimen was blinded throughout the entire analysis.

### Real-time PCR and sequence analysis

Six overlapping primer sets that cover the entire ORF for *Mycobacterium tuberculosis *complex sodA (GenBank No. AF061030) were designed using Primer Express software (Applied Biosystems Inc., Foster City, CA) and are described in Table 1. Real-time PCR, performed with SYBR Green I double-stranded binding dye (Applied Biosystems), and dissociation curve analysis were carried out according to the manufacturer's protocol on the ABI Prism 7000 Sequence Detection System. The quality of DNA extracted from each clinical specimen was assessed by screening for human beta actin, using the primers F 5'-ACCGAGCGCGGCTACAG-3' and R 5'-CTTAATGTCACGCACGATTTCC-3'. Human beta actin was amplified from all 36 clinical specimens. For each analysis, two microliters of MTB H37Rv genomic DNA (Colorado State University), corresponding to ~10^4 ^genomic copies, was used as the positive control, five microliters of irradiated water as the negative control, and five microliters of template DNA from each clinical specimen. Samples whose dissociation curve peaks had a melting temperature within 1°C of TB DNA were selected for further analysis on a 3% TBE gel. Bands that electrophoresed within approximately 10 bp of TB DNA were excised and purified using the QIAquick Gel Extraction Kit (QIAGEN Inc., Valencia CA). Both strands of the DNA were directly sequenced by GenePass, Inc (Nashville, TN). Only sequences verified from the positive and negative strand of the amplicon were used for analysis. Alignments of sodA sequences from sarcoidosis patients were performed using the NCBI BLAST program.

**Table 1:** Sequences of primers used to amplify sodA^a^

Name		Primer sequence	Amplicon length (bp)	Nucleotide (ORF)/amino acid position
**Region 1**	F^b^	5' CCGTGGCCGAATACACCTT 3'	127	-167
	R	5' CATTGGCGCCCTTTACGTA 3'		
**Region 2**	F	5' GAGCTTCACCACAGCAAGCA 3'	127	76–202/26–67
	R	5' AAGCTAGATTCTTTTCGTTCAGCAA 3'		
**Region 3**	F	5' CGTCGCCAAACTCGAAGAG 3'	151	129–279/44–93
	R	5' GCCGGTGGGCTTGTCA 3'		
**Region 4**	F	5' GCCACGTCAATCACACCATCT 3'	232	215–446/73–149
	R	5' AAGTTCGTCTGGTGGTCGTAAAC 3'		
**Region 5**	F	5' GGGACACACTCGGCAACAA 3'	158	389–546/131–182
	R	5' CAAAACGCCTTGGCAAAGTC 3'		
**Region 6**	F	5' TTTACGACCACCAGACGAACTTC 3'	161	425–585/150–195
	R	5' CGCATACCGTGACTGCACAT 3'		

^a^Based on M. tuberculosis H37Rv sodA sequence (GenBank no. AF061030)

^b^F – forward, R – reverse

### Phylogenetic analysis and sequence alignments

Sequences of sodA from various *Mycobacterium *species were obtained from GenBank. Because there is no genetic variability of sodA among members of MTB complex; sequence alignment was based upon GenBank no. AF061030. In addition to MTB complex and sarcoidosis amplicons, multiple corresponding nucleotide sequence alignments of sodA region 4 (base pairs 215–446, GenBank no. AF061030) from pathogenic and nonpathogenic mycobacteria were created using ClustalX. Phylograms of nucleotide alignments were generated using the Paup 4.0b10 neighbor-joining method, based on HKY85 distances. The resulting phylograms were displayed using midpoint rooting.

### Preparation of peripheral blood mononuclear cells (PBMC)

PBMC were isolated from blood drawn into tubes containing EDTA, and separated by Ficoll-Hypaque density gradient separation (Amersham Biosciences) according to the manufacturer's instructions. The PBMC were cryopreserved in fetal calf serum with 10% dimethy sulfoxide (DMSO), and stored in liquid nitrogen until time for analysis.

### Synthesis of mycobacterial peptides

Forty sodA peptides, 15-mers overlapping by 10, which span the entire amino acid sequence of MTB superoxide dismutase (GenBank no. AAD15824.1) were generated (Table 2). Each sodA peptide was synthesized by solid-phase F-moc chemistry (Genemed Synthesis, San Diego, CA), to a purity of >70%.

**Table 2:** SodA peptide sequences used in immune recognition assays

**Peptide**	**Position**	**Peptide sequence**	**Peptide**	**Position**	**Peptide sequence**
1	1–15	VAEYTLPDLDWDYGA	21	101–115	DAFGSFDKFRAQFHA
2	6–20	LPDLDWDYGALEPHI	22	106–120	FDKFRAQFHAAAT TV
3	11–25	WDYGALEPHISGQIN	23	111–125	AQFHAAAT TVQGSGW
4	16–30	LEPHISGQINELHHS	24	116–130	AAT TVQGSGWAALGW
5	21–35	SGQINELHHSKHHAT	25	121–135	QGSGWAALGWDTLGN
6	26–40	ELHHSKHHATYVKGA	26	126–140	AALGWDTLGNKLLIF
7	31–45	KHHATYVKGANDAVA	27	131–145	DTLGNKLLIFQVYDH
8	36–50	YVKGANDAVAKLEEA	28	136–150	KLLIFQVYDHQTNFP
9	41–55	NDAVAKLEEARAKED	29	141–155	QVYDHQTNFPLGIVP
10	46–60	KLEEARAKEDHSAIL	30	146–160	QTNFPLGIVPLLLLD
11	51–65	RAKEDHSAILLNEKN	31	151–165	LGIVPLLLLDMWEHA
12	56–70	HSAILLNEKNLAFNL	32	156–170	LLLLDMWEHAFYLQY
13	61–75	LNEKNLAFNLAGHVN	33	161–175	MWEHAFYLQYKNVKV
14	66–80	LAFNLAGHVNHT IWW	34	166–180	FYLQYKNVKVDFAKA
15	71–85	AGHVNHT IWWKNLSP	35	171–185	KNVKVDFAKAFWNVV
16	76–90	HT IWWKNLSPNGGDK	36	176–190	DFAKAFWNVVNWADV
17	81–95	KNLSPNGGDKPTGEL	37	181–195	FWNVVNWADVQSRYA
18	86–100	NGGDKPTGELAAAIA	38	186–200	NWADVQSRYAAATSQ
19	91–105	PTGELAAAIADAFGS	39	191–205	QSRYAAATSQTKGLI
20	96–110	AAAIADAFGSFDKFR	40	193–207	RYAAATSQ TKGLIFG

### ELISPOT analysis for interferon gamma production

ELISPOT assays were performed as previously described [15]. Briefly 96-well polyvinylidene difluoride-backed plates were coated with anti-interferon-γ [IFN-γ] mAb, 1-DIK (0.5 μg/ml; Mabtech, Stockhlm, Sweden) at 4°C overnight. The following day, PBMC were added directly at 10^5 ^cells/well in R10 media (RPMI-1640 supplemented with 2 mM L-glutamine, 50 IU/ml penicillin, 50 μg/ml streptomycin, and 10% heat inactivated FBS (Invitrogen Corporation, Irvine, CA) and then sodA peptides were added to individual wells in duplicate (20 μg/ml final concentration). Phytohemagluttin A (PHA) or media alone served as positive and negative controls, respectively. The plates were incubated for 18 hours at 37°C in 5% CO_2_. After washing six times with phosphate buffered saline (PBS) plates were incubated for 2 hours with biotinylated anti-IFN-γ mAb, 7-B6-1 (0.5 μg/ml; Mabtech). After additional washes, a 1:2,000 dilution of streptavidin-alkaline phosphatase conjugate was added to each well for 2 hours. The plates were then washed, and IFN-γ producing cells detected after a 10–20 min color reaction using chromogenic alkaline phosphatase substrate (BCIP/NBT Substrate Kit, Vector Laboratories). Interferon gamma (IFN-γ) producing cells were counted using an IMMUNOSPOT 3 Analyzer (Cellular Technology Limited (C.T.L); Cleveland, OH). Results were expressed as the number of spot-forming cells (SFC) per 10^6 ^PBMC. The mean number of spots in duplicate negative control wells was less than two for all 26 PPD- participants. A positive response was defined as at least 50 SFC/10^6 ^PBMC, and at least three times above background. The definition of a positive result was based upon a prior analysis, involving recognition of ESAT-6 and katG in systemic sarcoidosis [9]. Assays using PBMC from PPD-, PPD+ and sarcoidosis subjects were performed simultaneously; the technicians were blinded to the clinical diagnosis of each study participant throughout the analysis.

### Statistical Analysis

Comparisons of the distribution of T cell frequencies were performed using the Wilcoxon rank sum test. Categorical comparisons, such as the percentage of individuals with immune reactivity to mycobacterial antigens, were made using Fisher's exact test. All performed comparisons are reported, all p-values are 2-sided, and all analyses were performed using R (Version 2.1.1).

## Results

### Clinical characteristics of study participants

Of the 17 sarcoidosis subjects, 5(29%) were African-American, 9(53%) were male, and 16(94%) were ≤ 50 years of age (Table 3). Biopsies were taken from lung, lymph node, or skin. Of the 16 control subjects, 1(6%) was African-American, 9(56%) were male, and 11(69%) were ≤ 50 years of age. Biopsies were taken from lung, lymph node, skin or colon. Of the three tuberculosis patients, one was African-American, all were male and <50 years of age (Table 3). Biopsies were taken from lymph node in two patients, and the meniscus of the knee of the third patient. Acid-fast bacilli (AFB) were highly visible in two specimens (TB 1 and 3) and rare in one specimen (TB 2); *M. tuberculosis *was cultured from all three. Granulomatous involvement was noted in all 17 sarcoidosis specimens, 10 of 16 control specimens, and all three tuberculosis specimens (Table 3). Ten of the 17 sarcoidosis subjects had undergone tuberculin skin testing at the discretion of their primary physician; all ten were negative, as were all of the PPD- healthy volunteers. The status of the remaining seven is unknown.

**Table 3:** Molecular analysis for sodA among sarcoidosis and control specimens

**Patient**	**Age, Sex, Race**^a^	**Biopsy site**	**Pathologic diagnosis**	**Presence of granuloma**	**Tissue storage**^b^	**PCR analysis**^c^	**sodA region(s)**
Sarcoid 1	31, M, C	Lung	Sarcoidosis	Yes	Frozen	Mtb complex	4, 5
Sarcoid 2	46, F, C	Lymph node	Sarcoidosis	Yes	Frozen	GDM	4
Sarcoid 3	65, M, U	Lymph node	Sarcoidosis	Yes	Frozen	Mtb complex	2, 4
Sarcoid 4	29, F, C	Lymph node	Sarcoidosis	Yes	FFPE	-	-
Sarcoid 5	36, F, AA	Lung	Sarcoidosis	Yes	Frozen	Mtb complex	4
Sarcoid 6	48, F, AA	Skin	Sarcoidosis	Yes	FFPE	Mtb complex	3
Sarcoid 7	30, M, AA	Skin	Sarcoidosis	Yes	Frozen	Mtb complex	5
Sarcoid 8	33, F, C	Lymph node	Sarcoidosis	Yes	FFPE	Mtb complex	4
Sarcoid 9	50, F, AA	Lymph node	Sarcoidosis	Yes	FFPE	-	-
Sarcoid 10	50, M, C	Lymph node	Sarcoidosis	Yes	FFPE	Mtb complex	5
Sarcoid 11	41, F, C	Lymph node	Sarcoidosis	Yes	FFPE	-	-
Sarcoid 12	39, M, C	Lymph node	Sarcoidosis	Yes	FFPE	-	-
Sarcoid 13	46, M, AA	Lymph node	Sarcoidosis	Yes	FFPE	Mtb complex	5
Sarcoid 14	45, M, C	Skin	Sarcoidosis	Yes	FFPE	Mtb complex	2
Sarcoid 15	34, F, C	Lymph node	Sarcoidosis	Yes	FFPE	GDM	4
Sarcoid 16	28, M, C	Lymph node	Sarcoidosis	Yes	Frozen	-	-
Sarcoid 17	34, M, C	Lymph node	Sarcoidosis	Yes	FFPE	Mtb complex	2
Control 1	70, F, C	Colon	Normal colon	No	Frozen	-	-
Control 2	22, F, C	Lymph node	Histoplasmosis	Yes	Frozen	Mtb complex	4, 5
Control 3	60, M, C	Lymph node	Nocardia	Yes	Frozen	-	-
Control 4	56, F, C	Lymph node	Cholangial carcinoma	No	Frozen	-	-
Control 5	49, M, C	Lymph node	Nonspecific Interstitial Pneumonia	No	Frozen	-	-
Control 6	11, F, AA	Skin	Blastomycosis	Yes	FFPE	-	-
Control 7	51, F, C	Lung	Histoplasmosis	Yes	FFPE	-	-
Control 8	26, M, C	Lung	Histoplasmosis	Yes	FFPE	-	-
Control 9	30, M, C	Lung	Blastomycosis	Yes	FFPE	-	-
Control 10	25, M, C	Skin	Blastomycosis	Yes	FFPE	-	-
Control 11	26, M, C	Skin	Blastomycosis	Yes	FFPE	-	-
Control 12	36, M, U	Lung	Hodgkin's lymphoma	No	Frozen	-	-
Control 13	15, M, U	Lymph node	Hodgkin's lymphoma	No	Frozen	-	-
Control 14	68, M, C	Lymph node	Sinus histiocytosis	No	Frozen	-	-
Control 15	47, F, C	Lymph node	Histoplasmosis	Yes	FFPE	-	-
Control 16	35, F, C	Lung	Cryptococcus	Yes	FFPE	Mtb complex	4
TB 1	30, M, C	Lymph node	Tuberculosis	Yes	FFPE	Mtb complex	3, 5
TB 2	46, M, C	Knee	Tuberculosis	Yes	FFPE	Mtb complex	2, 4, 5
TB 3	36, M, H	Lymph node	Tuberculosis/AIDS	Yes	FFPE	Mtb complex	1 – 6

^a^C-Caucasian, AA-African American, H-Hispanic, U-Unknown; ^b^FFPE – formalin-fixed, paraffin-embedded ^c^GDM – genetically distinct mycobacteria

### Sequences consistent with MTB complex sodA were amplified from sarcoidosis granulomas

Multiple regions of sodA were amplified from all three tuberculosis patients (Table 3). The number of regions that we were able to amplify correlated with the number of bacilli seen on histologic staining (Table 3). For example, we amplified all six regions of MTB complex sodA from TB 3, a lymph node from an AIDS patient, containing numerous AFB. In contrast, we amplified only three regions from TB 2, a knee specimen, which possessed only rare AFB on histologic stain. Each sarcoidosis sample was AFB negative, and we generally amplified a single region of sodA, compared to multiple ones. Due to the small number of TB specimens tested, it is difficult to determine a true correlation of amplication cycle and detection of mycobacteria by AFB histologic staining. The true correlation will require testing additional MTB specimens.

**Figure 1:**
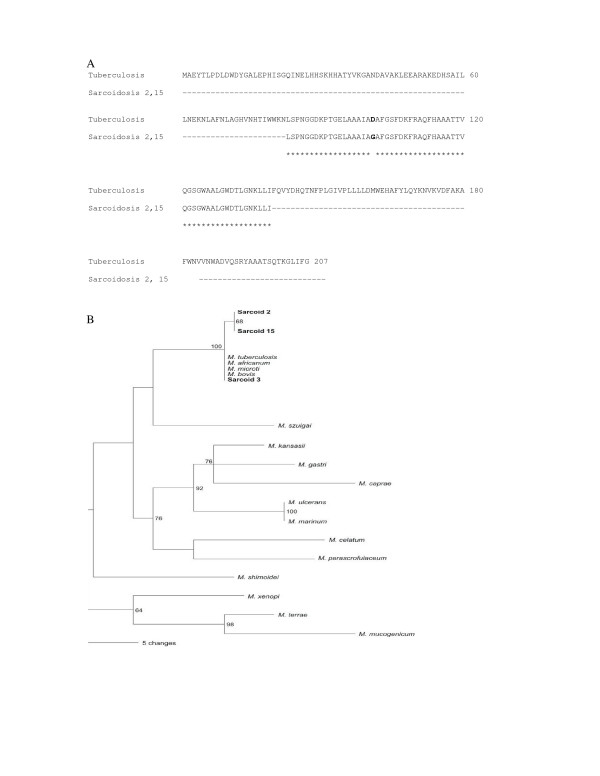
**Comparison of sodA amplicons generated from sarcoidosis granulomas to *M. tuberculosis *sodA**. PCR analysis for sodA nucleic acids in sarcoidosis granulomas revealed amplicons with 100% positional identity with *M. tuberculosis *complex (MTB) among 10 of 12 sarcoidosis specimens, represented by Sarcoidosis 3. Region 4 of Sarcoid 2 contained the same nucleotide substitution as Sarcoid 15 (A302G), resulting in the amino acid substitution D101G (Figure 1A). The two sarcoidosis samples were processed three months apart. Phylogenetic analysis of the amplicons placed all the sequences as most consistent with members of MTB complex, but noted that the sequences detected in Sarcoid 2 and 15 were distinct from other members, including the 10 sarcoidosis samples (Figure 1B).

SodA amplicons were detected in 12 of 17 sarcoidosis specimens, compared to two of 16 negative control specimens (p = 0.001) (Table 3). The sarcoidosis amplicons spanned regions 2–5; regions 1 and 6 were not amplified from any of the sarcoidosis specimens. Six specimens were positive for region 4 and four for region 5. In Sarcoid 1, amplicons from regions 4 and 5 were detected, resulting in a segment of sodA that was 276 bp long (GenBank accession no. DQ822576); in Sarcoid 3, regions 2 and 4 were detected. Sequence analysis of the amplicons from 10 specimens revealed 100% positional identity with MTB complex. The primers of regions 1 and 4 were capable of amplifying sodA nucleic acids from nontuberculosis mycobacteria, such as *M. kansasii *(personal observation). However, only sequences consistent with MTB complex were detected. In two specimens (Sarcoid 2 and 15), the amplicons from region 4 contained 99% positional identity with MTB complex. The same polymorphism, A302G, was detected in both specimens (GenBank accession no. DQ768096), despite these samples being processed and analyzed three months apart. This nucleotide substitution resulted in a D101G mutation (GenBank accession no. ABG68026) in a previously published amino acid sequence for MTB sodA (GenBank accession no. AAD15824.1) (Figure 1A). Phylogenetic analysis of the unique sarcoidosis sodA sequences at position 215–446 based upon GenBank No. AF061030 (Region 4) distinguished them from other members of MTB complex (Figure 1B).

It has been reported that environmental mycobacteria preferentially localize to established tuberculous granulomas [16]. In order to determine if the detection of mycobacterial sodA in sarcoidosis granulomas reflected preferential localization of mycobacteria to granulomatous inflammation, we analyzed granulomatous controls of known etiology. Despite extracting and analyzing sarcoidosis and controls specimens simultaneously, sodA nucleic acids were absent in all but 2 control patients, Controls 2 and 16. MTB complex sodA amplicons were detected in these two specimens, although they were negative for mycobacteria by both histology and culture. In Control 2, sodA amplicons were detected for regions 4 and 5, while only region 4 was amplified from Control 16. There was a significant difference for the detection of sodA nucleic acids among the granulomatous sarcoidosis specimens (12 of 17), in comparison to the granulomatous negative controls (2 of 10) (p = 0.018) (Table 3).

Frozen and paraffin-embedded specimens sarcoidosis and control specimens were included to determine whether mycobacteria were introduced during tissue fixation. There was not a significant difference in the presence of sodA nucleic acids among the sarcoidosis specimens that were frozen in comparison to those that were formalin-fixed, paraffin-embedded (5/6 versus 7/11, p = 0.6) (Table 3).

### Immune recognition of sodA is distinct between sarcoidosis and PPD- healthy volunteers

Several reports have demonstrated the presence of cellular immune responses to mycobacterial antigens among patients infected with MTB [12,17]. Because analysis of the sarcoidosis amplicons were most consistent with MTB complex sodA, we generated 40 peptides that cumulatively span the entire MTB sodA protein (GenBank no. AAD15824.1), and stimulated PBMC from a subgroup of sarcoidosis, PPD- and PPD+ subjects to identify immunogenic peptides. There was no recognition of any of the 40 sodA peptides among the PPD- control subjects. Among the sarcoidosis and PPD+ subjects, four of the 40 peptides were recognized most frequently: sodA 31, 33, 36 and 38 (Table 2). PBMC from all 49 subjects were tested against these four peptides; peptides before or after peptides 31, 33, 36 and 38 were also tested if PBMC were available.

Of the 17 sarcoidosis subjects from whom we obtained archival tissues for molecular analysis, we were able to obtain PBMC from 12. The clinical characteristics of the sarcoidosis subjects were detailed earlier. Because the biopsy and PBMC were not obtained simultaneously, there was variation in the age of the sarcoidosis subjects (Table 3 and 4 reflects the subject's age at acquisition of tissue or PBMC, respectively). In addition to these 12 subjects, we obtained PBMC from 26 PPD- healthy volunteers, and 11 subjects with PPD+. Of the 26 PPD- patients, 14(54%) were African American, 6(23%) were male and 20(77%) ≤ 50 years of age (Table 4). Of the 11 PPD+ subjects, 4(36%) were African American, 3(27%) male, and 8(73%) ≤ 50 years of age (Table 4).

**Table 4:** Immune recognition to sodA peptides among study participants

**Subject**	**Age**^**a**^**/Sex**^**b**^**/Race**^**c**^	**Site of Involvement**^**d**^	sodA*	**Subject**	**Age**^**a**^**/Race**^**b**^**/Sex**^**c**^	**Site of Involvement**^**d**^	sodA*
**Sarcoidosis 6**	50, F, AA	P, C	**70**	**Control 1**	43, F, AA	None	0
**Sarcoidosis 7**	31, M, AA	CNS, P, C	**150**	**Control 2**	38, F, C	None	0
**Sarcoidosis 8**	34, F, C	P	**100**	**Control 3**	27, F, AA	None	10
**Sarcoidosis 9**	55, F, AA	P	**65**	**Control 4**	33, F, AA	None	0
**Sarcoidosis 10**	56, M, C	P	0	**Control 5**	23, M, C	None	0
**Sarcoidosis 11**	47, F, C	P, CNS	0	**Control 6**	25, F, C	None	0
**Sarcoidosis 12**	42, M, C	P	0	**Control 7**	31, M, AA	None	0
**Sarcoidosis 13**	51, M, AA	P	**110**	**Control 8**	52, F, C	None	0
**Sarcoidosis 14**	45, M, C	P, C	0	**Control 9**	32, F, C	None	0
**Sarcoidosis 15**	34, F, C	P	0	**Control 10**	34, F, AA	None	0
**Sarcoidosis 16**	30, M, C	P	**160**	**Control 11**	29, M, C	None	0
**Sarcoidosis 17**	33, M, C	P	0	**Control 12**	52, F, C	None	0
				**Control 13**	35, F, C	None	0
				**Control 14**	57, F, C	None	0
				**Control 15**	54, F, C	None	0
**PPD+ 1**	49, F, C	Latent	**360**	**Control 16**	57, F, C	None	10
**PPD+ 2**	50, F, AA	Latent	**50**	**Control 17**	55, F, AA	None	0
**PPD+ 3**	42, F, AA	Latent	**100**	**Control 18**	37, M, AA	None	0
**PPD+ 4**	58, M, AA	Latent	**65**	**Control 19**	44, F, C	None	0
**PPD+ 5**	50, F, C	Latent	**50**	**Control 20**	32, F, AA	None	0
**PPD+ 6**	60, F, AA	Latent	0	**Control 21**	32, F, AA	None	**500**
**PPD+ 7**	38, F, C	Latent	0	**Control 22**	27, F, AA	None	0
**PPD+ 8**	49, F, C	Latent	0	**Control 23**	42, F, AA	None	0
**PPD+ 9**	42, M, AA	Latent	**55**	**Control 24**	32, M, AA	None	0
**PPD+ 10**	51, F, C	Latent	0	**Control 25**	26, F, AA	None	0
**PPD+ 11**	30, M, C	Latent	0	**Control 26**	50, M, AA	None	0

^a^Age in years; ^b^AA, African-American; C, Caucasian; ^c^F, female, M, male; ^d ^P, Pulmonary; C, Cutaneous; CNS, Central Nervous System; ABD, Abdominal; *Inteferon-γ producing spot-forming cells per million PBMC

Using the ELISPOT assay, we detected immune recognition of sodA peptides among six of 12 sarcoidosis subjects, compared to one of the 26 PPD- controls (p = 0.002), and six of 11 subjects with PPD+ infection (p = 1.0) (Table 4). We performed multivariable logistic regression analysis to assess if factors such as sex, race, or site of involvement affected recognition of sodA. There was no significant association with either of these factors, although there was a trend toward significance in regards to race. For example, 4/4 African-Americans (AA) with sarcoidosis recognized sodA using ELISPOT, compared to 2/8 Caucasians (p = 0.06); 4/5 PPD+ AA recognized sodA compared to 2/6 Caucasians (p = 0.24); and 1/14 PPD- control AA recognized sodA compared to 0/12 Caucasians (p = 1.0). Although none of these comparisons are statistically significant, this could be due to low power. In fact, we performed multivariable logistic regression, adjusting for race (AA or non-AA) and group (PPD- control, sarcoidosis, or PPD+), and AA race was significantly associated with increased recognition (p = 0.01). However, after adjusting for race, sarcoidosis and PPD+ participants were still more likely to recognize sodA than PPD- controls (p = 0.002 and 0.01, respectively), consistent with the unadjusted analyses.

**Figure 2:**
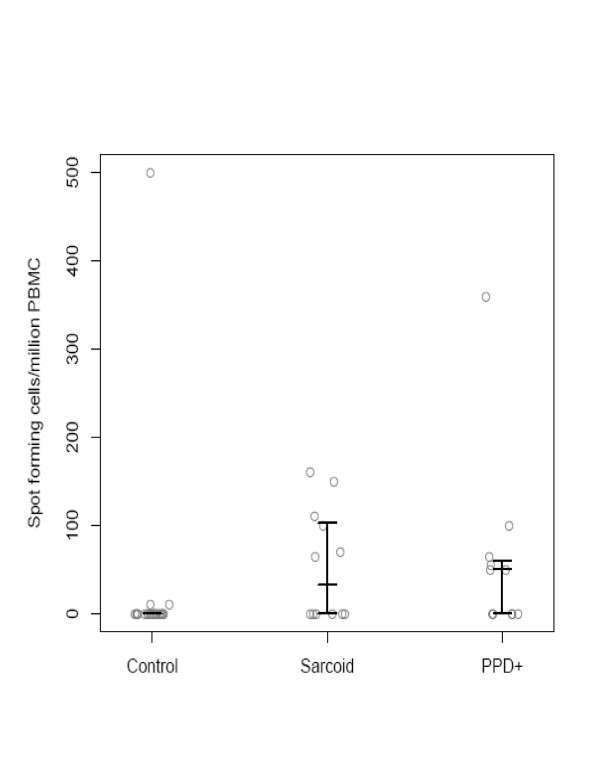
**Distribution of T cell frequencies among PPD negative, sarcoidosis and PPD+ subjects**. The horizontal bars represent the 25^th^, 50^th ^and 75^th ^percentile respectively. There was a lack of recognition of sodA peptides among the majority of the PPD- control group, and a significant difference overall among the three groups. Among the sarcoidosis subjects, the distribution of the T cell frequencies among the sarcoidosis subjects more closely paralleled that of the PPD positive subjects also, rather than the PPD negative healthy volunteers.

**Figure 3:**
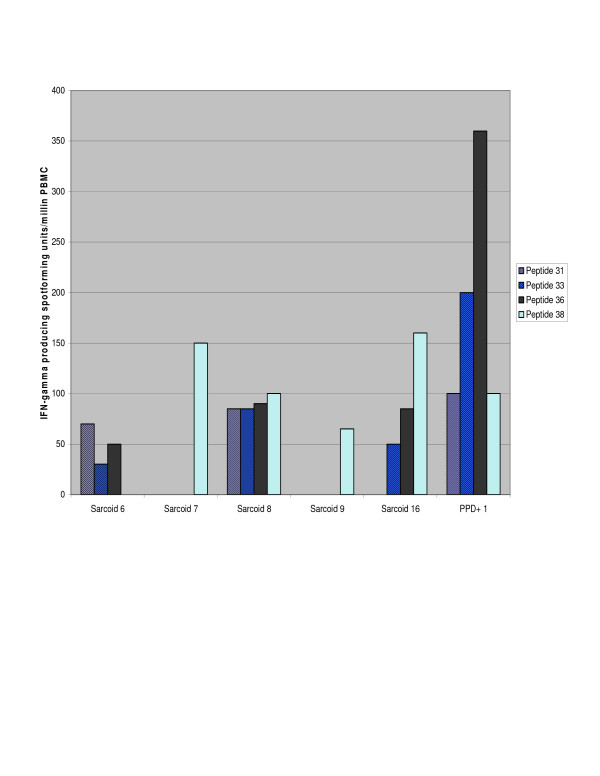
**Recognition of multiple sodA peptides among individual sarcoidosis subjects**. Peptide screening identified peptides toward the terminal end of sodA as being immunogenic. Among the sarcoidosis subjects who recognized sodA, recognition of more than one peptide was frequently observed. Peptides 36 and 38 were frequently immunogenic, although there was variation in the magnitude of the response generated by each subject. Numerous sodA peptides were recognized by PPD+ subjects. A representative analysis of the PPD+ subjects is included. The recognition of multiple sodA peptides by the sarcoidosis subjects suggests that the sarcoidosis Th-1 immune response may be elicited by multiple antigenic peptides rather than a single dominant antigen.

Despite negative culture and histology for mycobacterial infection in sarcoidosis specimens, there was a significant difference in recognition of sodA peptides between the sarcoidosis and PPD- healthy volunteers, but not between sarcoidosis and PPD+ subjects. We also assessed the distribution of the T cell frequencies among the PPD-, sarcoidosis and PPD+ subjects. There was a significant difference between the sarcoidosis and PPD- subjects (p = 0.002, Wilcoxon), but again, not between the sarcoidosis and PPD+ subjects (p = 0.72) (Figure 2). Three sarcoidosis subjects recognized two or more sodA peptides, demonstrating that multiple sodA epitopes generate a Th-1 immune response (Figure 3).

### The majority of sarcoidosis subjects have molecular or immunologic evidence of *Mycobacterium *sodA

Prior PCR analysis and immune recognition assays for the presence of sodA was significantly distinct between the sarcoidosis and PPD- control subjects. We were interested in the number of sarcoidosis subjects possessing molecular or immunologic evidence for sodA. There were 12 sarcoidosis subjects of whom we obtained archival tissue and blood. Eight of the 12 were positive by PCR analysis, and six positive by the ELISPOT assay. Four subjects (Sarcoid 6–8, 13) were positive for sodA nucleic acids, as well as recognition of sodA peptides; Sarcoid 11 and 12 were negative by both assays (Table 5). In total among the 12 specimens, 10 were positive by PCR or by ELISPOT analysis and 50% of the specimens had molecular and immunologic agreement for the presence or absence of sodA (Table 5).

**Table 5:** Comparison of molecular and immunologic analysis for the detection of sodA.

	**PCR**	**ELISPOT**
Sarcoid 6	+	+
Sarcoid 7	+	+
Sarcoid 8	+	+
Sarcoid 9	-	+
Sarcoid 10	+	-
Sarcoid 11	-	-
Sarcoid 12	-	-
Sarcoid 13	+	+
Sarcoid 14	+	-
Sarcoid 15	+	-
Sarcoid 16	-	+
Sarcoid 17	+	-

+, positive analysis for sodA; -, negative analysis for sodA

## Discussion

The absence of histologic and microbiologic evidence of microorganisms in the sarcoidosis granulomas has led to questions about a role for infectious agents in sarcoidosis immunopathogenesis. This report describes molecular and immunologic support for the presence of the virulence factor, *Mycobacterium *sodA, in systemic sarcoidosis.

The strength of molecular analysis for sodA lies in the ability to identify pathogenic *Mycobacterium *species [13]. The detection of sodA nucleic acids yielded important information in this study. Although the primers in region 1 and region 4 were capable of detecting nontuberculous mycobacteria, sequence analysis of the amplicons was most consistent with members of MTB complex, thus revealing mycobacteria capable of inducing granuloma formation. The majority of the sequences possessed 100% positional identity with MTB (GenBank No. AF061030); however, sequence analysis of the amplicons also revealed sequences that were genetically distinct (Table 3, Figure 1B). To confirm a polymorphism, we insured that the nucleotide substitution was present on both strands and that an actual peak was visualized on the chromatogram. The detection of the polymorphism, A302G, in two sarcoidosis specimens processed and analyzed at separate time points also suggests that this polymorphism is not due to PCR error.

Phylogenetic analysis of the amplicons derived from the sarcoidosis granulomas revealed three major points: 1) There is a clear association between the sarcoidosis amplicons and the sequences of members of MTB complex, demonstrating that they share a recent common ancestor, and precluding the possibility that the sarcoidosis sequences are derived from distantly related mycobacterial or Nocardia species. 2) The sequences from Sarcoidosis 2 and 15 form a distinct clade, emphasizing that these sequences may belong to a single mycobacterial species (Figure 1B). Whether these mutations reflect a distinct member of MTB complex or *M. tuberculosis *strain with a mutation in sodA is unclear. A sodA mutation, unique from that found in the two sarcoidosis specimens, has been described in an isoniazid-resistant MTB strain [18]. 3) The substantial evolutionary distances between the sarcoidosis amplicons and the atypical mycobacteria eliminate the possibility that we have sequences of environmental mycobacteria as a contaminant.

Superinfecting mycobacteria preferentially home to tuberculous granulomas [16]. This observation has been used to explain the detection of mycobacterial nucleic acids in sarcoidosis granulomas. We included in this analysis 10 granulomatous control specimens of known etiology. SodA DNA was detected in only two of them. One of the specimens was a lymph node containing *Histoplasma capsulatum *in an individual with rheumatoid arthritis. Prior reports have noted an association with latent tuberculosis in such patients [19]. The detection of mycobacterial sodA in sarcoidosis specimens in a significantly different proportion compared to granulomatous negative control specimens suggests that the presence of sodA sequences does not simply reflect preferential localization of environmental mycobacteria to established granulomas.

The inclusion of sarcoidosis and control specimens that were frozen or paraffin-embedded was designed to address the possibility that mycobacteria are introduced into sarcoidosis specimens during tissue fixation. There are reports of environmental mycobacteria in hospital water systems, and well as fiberoptic bronchoscopes [20,21]. We chose snap frozen and paraffin-embedded tissue specimens among the sarcoidosis and PPD- controls specimens. The absence of sodA in the majority of the negative control specimens, as well as the presence of sodA nucleic acids in snap frozen and paraffin-embedded sarcoidosis tissues, suggests that the mycobacterial nucleic acids detected are not present due to contamination during tissue fixation. Identification of immunogenic sodA peptides has not been previously reported in tuberculosis or sarcoidosis subjects. Despite the absence of mycobacteria by histologic staining and culture of sarcoidosis specimens, the immune response to sodA among the sarcoidosis subjects more closely reflected the response among PPD+ subjects than PPD- volunteers. There was not a significant difference between the sarcoidosis and tuberculosis subjects in the percentage of subjects recognizing these peptides or the distribution of the T cell frequencies (Figure 2). Many of the sarcoidosis and PPD+ subjects recognized more than one peptide, suggesting that the immune response observed in these subjects can be elicited by multiple sodA epitopes (Figure 3). Due to limitations in the number of PBMC, we were unable to test all 40 sodA peptides, so it is possible that sodA epitopes other than the four that we tested exist.

The dual assessment for mycobacteria in sarcoidosis pathogenesis provides interesting information. Among the 12 subjects, 10 possessed molecular or immunologic evidence for sodA. This illustrates the importance of using complementary approaches to assess for microorganisms in pathologic specimens of unknown etiology. The two sarcoidosis subjects who were negative by both methods suggest that mycobacteria do not have a role in all sarcoidosis specimens, supporting other reports of the inappropriate diagnosis of sarcoidosis in subjects with disease with close phenotypic and pathologic features, such as chronic beryllium disease [22]. Thus sarcoidosis may be a common pathologic phenotype that can be caused by infectious or non-infectious etiologies.

PCR of archival specimens was more sensitive for sodA than ELISPOT analysis of peripheral blood (Table 5). There was a time lapse between acquisition of the blood and tissue samples in a given patient; this differential timing of biopsies and blood testing is a major limitation of the analysis. The common observance in the dual analysis was a positive PCR analysis for sodA but a lack of recognition by ELISPOT analysis. This result is likely due to the preservation of nucleic acids by tissue fixation, as opposing to an evolving host immune response. Some of the patients had resolved their disease between the diagnostic biopsy and acquisition of PBMC. Concordantly, there exists evidence that immune recognition of mycobacterial antigens cease with clearance of mycobacteria [17,23,24]. Future studies using blood and tissue of sarcoidosis subjects obtained simultaneously will be performed. Another limitation is that the PCR involves analysis of a site of active sarcoidosis involvement [ie the granuloma], whereas the immunologic analysis involves systemic responses. Sensitivity for immune recognition might improve by using lymphocytes from sites of active inflammation such as bronchoalveolar lavage fluid or directly from lymphocytes derived from fresh sarcoidosis granulomas. Finally, the sensitivity of the ELISPOT may have improved if we had tested all 40 sodA peptides. It is unlikely that the four peptides are recognized by all HLA types [25].

## Conclusion

The most interesting finding in this study was the demonstration that molecular analysis of sarcoidosis granulomas for microbial virulence factors can lead to identification of target antigens. The responses detected closely parallel prior reports of immune recognition of secreted proteins in mycobacterial infections [17,26]. A dual analysis for microbial nucleic acids and immune responses increases the sensitivity for detecting microbial virulence factors in idiopathic diseases, providing complementary mechanisms.

## Competing interests

The authors declare that they have no competing interests.

## Authors' contributions

SSA carried out the molecular genetic studies, and performed the sequence alignment. WE determined immunogenic sodA epitopes and carried out the immunoassays, along with RH, MN, and JC. DTP performed the phylogenetic analysis and helped draft the manuscript. BES performed the statistical analysis. JEJ confirmed the pathologic diagnosis of all samples. WPD conceived of the study, participated in its design and coordination, and drafted the manuscript. All authors read and approved the final manuscript.

## Consent

This study was approved by the Vanderbilt University Institutional Review Board for human studies, and informed written consent was obtained from the study participant or their surrogates, if required. A copy of the written consent is available for review by the Editor-in-Chief of this journal at any time.
